# Inter-brain ERPs alignment during a joint Simon task: An EEG hyperscanning study

**DOI:** 10.1371/journal.pone.0338934

**Published:** 2026-01-08

**Authors:** Francesca Miti, Jlenia Toppi, Angela Ciaramidaro, Laura Astolfi, Cristina Iani, Sandro Rubichi

**Affiliations:** 1 Department of Biomedical, Metabolic and Neural Sciences, University of Modena and Reggio Emilia, Reggio Emilia, Italy; 2 Department of Computer, Control and Management Engineering A. Ruberti, Sapienza University of Rome, Rome, Italy; 3 IRCCS Fondazione Santa Lucia, Rome, Italy; 4 Center of Neuroscience and Neurotechnology, University of Modena and Reggio Emilia, Modena, Italy; 5 Department of Surgery, Medicine, Dentistry and Morphological Sciences with Interest in Transplant, Oncology and Regenerative Medicine, University of Modena and Reggio Emilia, Reggio Emilia, Italy; Universite Clermont Auvergne, FRANCE

## Abstract

The joint Simon task (JST) is widely employed to study the neurocognitive mechanisms underlying joint actions. Behavioral and electrophysiological research using this task suggests that individuals integrate their partners’ actions into their cognitive representations during collaborative activity, a concept referred to as the co-representation hypothesis. A key open question is whether this co-representation is accompanied by inter-brain synchronization. In this study, we investigated inter-brain dynamics in pairs of interacting participants by recording scalp electrophysiological (EEG) activity from 88 individuals performing the JST in dyads, using an EEG hyperscanning setup. We calculated the EEG-JSE, which represents the difference in ERP peak latencies between corresponding and non-corresponding trials, as an index of the neural joint Simon effect. This analysis focused on two ERP components, N2 and P3, which have been associated with the inhibition of response preparation and execution, respectively—processes that are crucial to joint Simon task performance. Furthermore, we examined whether the EEG-JSEs of the two participants in each pair were synchronized. Our findings revealed temporal alignment between the responding and non-responding participants in the pair, highlighting the unique inter-brain interaction dynamics that arise from the demands of performing a task jointly.

## Introduction

The ability to efficiently interact with other people has played a crucial role in human survival and evolution [1], to the extent that the human brain seems to have been deeply shaped by the need to deal with the complexity of a social environment, as clearly shown by the ability to automatically represent others [2,3].

**Fig 1:**
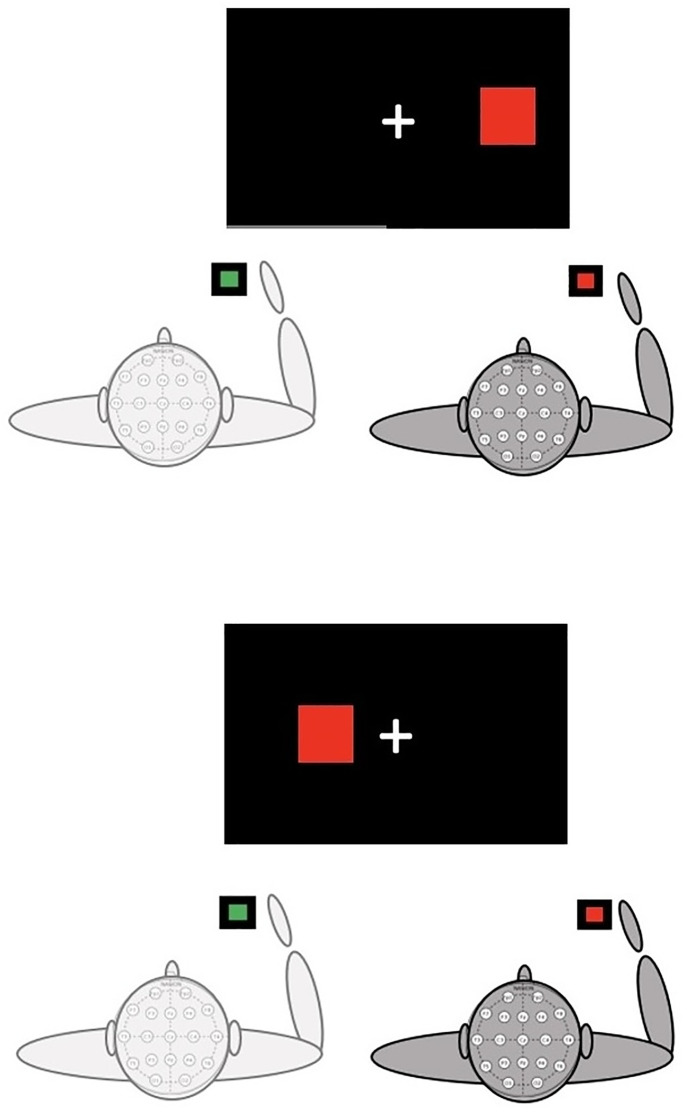
The Joint Simon task. Illustration of a corresponding (upper panel) and a non-corresponding (lower panel) trial in the Joint Simon task. In corresponding trials, the stimulus appears on the same side as the response, while in non-corresponding trials it appears on the opposite side. Only one of the two participants responds in each trial, hence a *go* trial for one participant is a *nogo* trial for the other. In the example depicted in the figure, the participant on the left responds to green stimuli, while the participant on the right responds to red stimuli. Since the stimulus is red, the trial is a *go* trial for the participant on the right and a *nogo* trial for the participant of the left. The participant in charge of responding (*go* trial) is represented in dark gray, while the participant who needs to refrain from responding (*nogo* trial) is represented in light gray. The correspondence level of the trial is always defined in relation to the responding participant.

Recent years have seen increased interest in the study of joint actions, which, as defined by Sebanz, Bekkering and Knoblich [4], refer to “any form of social interaction whereby two or more individuals coordinate their actions in space and time to bring about a change in the environment”. Crucially, empirical investigations have demonstrated that the presence of others affects our actions even in simple experimental tasks in which there is no need for coordination between the participants to perform efficiently [5]. An example of this is the Joint Simon Task (JST) [6,7] in which two individuals perform a choice reaction time (RT) task, each responding to one stimulus type. Specifically, the JST consists of the social version of the individual Simon task [8], which is a classic cognitive conflict task in which one participant is required to press a lateralized key in response to a non-spatial stimulus feature (e.g., color) while ignoring its left-right location. Typically, performance is faster and more accurate when stimuli and responses are on the same side (i.e., spatially corresponding trials) than when they are on opposite sides (i.e., spatially non-corresponding trials). This difference in performance between corresponding and non-corresponding trials is termed the Simon effect and is thought to reflect the conflict emerging at the response selection stage between two response codes: the response code generated based on task instructions and the one automatically activated by stimulus position [9,10]. Indeed, when individuals perform a *go-nogo* version of the task alone in which they are required to respond to only one stimulus type, and hence there is only one response alternative, the Simon effect is usually not observed, that is, RTs are not affected by whether the stimulus is on the same side or the opposite side of the response [11, 12]. Intriguingly, Sebanz et al. [6,7] showed that the Simon effect emerges when the task is shared between two individuals. In this arrangement, each participant responds only to one stimulus type using a single response key, effectively performing a *go-nogo* task (in which a *go* trial for one participant represents a *nogo* trial for the other; see Fig 1 for examples of trials in the joint Simon task). Nevertheless, participants’ responses are faster when the stimulus appears on their own side (i.e., corresponding trials) compared to when it appears on the other participant’s side (i.e., non-corresponding trials). This result, known as the Joint Simon Effect (JSE), has been explained by proposing that co-acting individuals represent each other’s part of the task and integrate it in their action planning (co-representation account, e.g., [6]). This process creates the preconditions for the cognitive conflict that underpins the Simon effect typically observed in the individual two-choice version of the task.

Notably, although Sebanz et al.’s [6] account has strong experimental support, alternative explanations suggest that the Joint Simon Effect (JSE) arises from spatial response coding [see 13 for a review]. These views converge on the idea that a co-actor serves as a salient reference point, offering an alternative spatial response [13–15]. Recently, the debate has shifted from social vs. non-social interpretations to viewing the JSE as a marker of self-other integration [16], reflecting how individuals represent and incorporate others’ actions during joint tasks [17]. The process of self-other integration is thought to reintroduce the stimulus-response conflict seen in the individual two-choice task.

Since behavioral investigations of the JSE reveal the impact of acting in a social context specifically on responding participants, researchers have increasingly focused on the electrophysiological activity associated with joint action performance that can be recorded for both paired participants. Their goal is to determine whether self-other integration processes are evident in modulations of two event-related potential (ERP) components: the N2 and the P3 [18–21]. The N2 is a negative ERP component that emerges approximately 200 milliseconds after stimulus presentation, with a peak at fronto-central electrodes [22,23]. This component has been linked to processes such as conflict monitoring, detection, and inhibition [24]. Notably, the N2 is particularly prominent in *nogo* trials [25] and in trials involving conflicting information, such as non-corresponding trials in interference tasks like the Simon task [26,27]. It is suggested that modulations in the N2 reflect the decision-making process of withholding a response rather than motor inhibition itself [26]. In contrast, the P3 is a positive component occurring 300–500 milliseconds after stimulus presentation [28]. In *go-nogo* tasks, the P3 exhibits a centro-parietal maximum in *g*o trials (*go* P3) and a fronto-central maximum in *nogo* trials (*nogo* P3). These subcomponents are thought to represent distinct processes: stimulus evaluation and response selection (*go* P3), and inhibitory mechanisms related to action control (*nogo* P3) [25,29].

Investigations of the JSE focused on these two components because the joint Simon task is thought to be characterized both by a conflict between task rules and response inhibition [19]. Indeed, in each trial, only one participant responds, and the execution of this response is associated with the need to inhibit a response by the other participant. In line with this view, Sebanz et al. [20] found a larger frontal *nogo* P3 in the social compared to the individual version of the task. These results were interpreted as an indication that in a joint task, since participants represent their co-actor task as if it was their own, they need to exert more action control in *nogo* trials to inhibit the tendency to respond [18,19,21]. Differently from Sebanz et al. [20] who found an effect of compatibility on the amplitude of the *go* P3 in both the individual and social contexts, Tsai et al. [21] found an effect of compatibility on the amplitude of the *nogo* P3 only in the social version of the task. More problematic are the results related to the N2 component. Indeed, while neither Sebanz et al. [20] or Tsai et al. [21] found a difference between the individual and the social versions of the task on the *nogo* N2, Ruissen & de Bruijn [19] found an increase in N2 amplitude for *go* trials in the social compared to the individual version of the Simon task in a group of participants administered with oxytocin, a neuropeptide which is known to increase social affiliation. They interpreted this finding by assuming that oxytocin enhanced task-sharing processes, hence increasing response conflict.

The EEG studies described above provide valuable insights by enabling the analysis of the effects of acting within a social context, even during *nogo* trials— those in which a behavioural response is unavailable. However, these studies have primarily focused on examining one brain at a time to compare joint and individual task conditions, aiming to identify modulations specific to the social context. In our view, a significant step forward in understanding the neural correlates of self-other integration processes would involve investigating inter-brain dynamics. Specifically, exploring the interactions between two brains during joint performance of the JST could offer a deeper understanding of the mechanisms underlying these processes. Recent improvements in signal recording and processing techniques applied to brain signals have successfully allowed the study of multiple-brain systems [30,31]. This approach, called *hyperscanning*, is based on the simultaneous recording and multivariate analysis of brain signals in pairs (or groups) of interacting subjects, allowing researchers to study neural synchronization during social interactions. Studies have shown that when people engage in activities such as conversation, cooperation, or joint decision-making, their brain waves tend to align, particularly in regions associated with social cognition and executive functions [32–34]. This inter-brain synchrony has been linked to improved communication, coordination, and mutual understanding [35–38]. In educational settings, neural coupling between teachers and students has been associated with more effective learning [39,40]. Additionally, hyperscanning EEG has been used to investigate social decision-making, joint action, and cooperation in ecological contexts, revealing distinct neural patterns underlying prosocial and competitive behaviors [41–44]. These findings suggest that brain-to-brain coupling is a fundamental mechanism shaping human interactions.

Within the hyperscanning approach, only few studies have explored the inter-brain dynamics during certain social games from the ERPs point of view [45,46], suggesting the possibility of an alignment of cognitive processes between interacting individuals which may be reflected in the inter-brain synchronization of ERPs. To the best of our knowledge, only a few studies used a hyperscanning approach to investigate the JSE*.* Specifically, three fNIRS studies co-registering pairs of participants during a JST demonstrated significant inter-brain neural synchronization in the inferior parietal lobe under both cooperative and competitive conditions [47], across the dorsolateral and medial parts of the prefrontal cortex in close interpersonal distances, such as with friends [48], and increased brain-to-brain synchrony at the temporal parietal junction (TPJ) under acute stress [49], hence validating the exploration of inter-brain connectivity during this kind of task. Still, no studies have assessed whether individuals show ERP inter-brain synchronization while performing the JST.

In the present study, we aimed to investigate the temporal inter-brain dynamics during the performance of the JST using the EEG hyperscanning approach. Specifically, we proposed that the temporal alignment of ERP components across pairs of participants can serve as a neural index of self-other integration. We hypothesize that, at the neural level, self-other integration processes underlying the JSE may be reflected in a temporal alignment of ERP latencies between the two participants, even though only one responds in each trial. To capture this effect, we introduced the EEG-JSE, defined as the difference in ERP peak latencies between corresponding and non-corresponding trials. By examining the EEG-JSE across paired participants, we aim to provide empirical evidence of inter-brain synchronization during joint task performance, further validating the use of ERP components to measure shared neural processes in cooperative settings.

Specifically, in the present study, forty-four couples performed a JST while the brain activity of each member of the couple was simultaneously recorded with an EEG hyperscanning setup. On a behavioural level, we expected to replicate the occurrence of a JSE effect, as reflected by longer RTs for non-corresponding than corresponding trials. As regards the ERP data, at the intra-brain level, we expected to replicate previous findings suggesting that *nogo* trials engage conflict detection and action control [20,21]. At the inter-brain level, we expected to find a synchronization between the EEG-JSE of the two participants. The significant added value of this measure is that, while behavioural responses are only available for the acting subject, and the classical ERP analyses shed light on the processes occurring in one single brain, the EEG hyperscanning approach gives us access to the neural counterpart of the JSE effect in both participants, enabling us to investigate also the non-acting participant’s neural activity occurring concurrently with that of the acting one, and to explore the potential temporal alignment between the two participants’ neural activity. Consequently, here we expect to find a synchronization of the EEG-JSE of the two participants in the couple even if only one member responded in each trial. By adding the measure of EEG-JSE to the tools available to highlight the neural mechanisms underlying interaction dynamics during JST performance, our findings aim to make a substantial contribution to the expanding literature on cooperative interpersonal interactions.

## Materials and methods

### Participants

Eighty-eight undergraduates (74 females, Mean Age: 22.4 years; SD: 6.5 years) with normal or corrected-to-normal vision participated in the study. The appropriate sample size to detect a main effect of Correspondence on RTs was calculated a-priori with G*Power 3.1 (effect size f = 0.17, α = 0.05, power = 0.95) [50]. The effect size was derived by the meta-analysis conducted by Karlinski, Lohse, and Lam [51]. The power calculation gave a recommended sample size of at least 76 participants.

To be included in the study, participants had to meet the following criteria: they were required to be over 18 years of age, right-handed, free from psychiatric or neurological disorders, and without a history of traumatic brain injury. Additionally, participants could not be using medications that might alter cortical excitability, such as antidepressants. They received course credit for their participation. Once selected, participants were asked to complete the Edinburgh Inventory [52] for the assessment of handedness by means of the laterality quotient (M = 0.75, SD = 0.25) and were randomly paired with another participant of the same sex.

All procedures were carried out in accordance with relevant guidelines and regulations of the University of Modena and Reggio Emilia and were in accordance with the Declaration of Helsinki. The study was approved by the Area Vasta Emilia Nord (AVEN) Ethics Committee (protocol n. 2019/0143036, study 1070/2019/SPER/ESTRE). Written informed consent was obtained from all participants. Study recruitment started on 10/01/2021 and ended on 05/19/2022.

### Apparatus and stimuli

The experimental session took place in a noiseless, dimly lit room. The two participants sat next to each other facing a 27’ screen at approximately 60 cm. A QWERTY keyboard located centrally with respect to the two participants was provided for key-press responses. The participant sitting on the left was assigned the ‘z’ key, while the ‘underscore’ key was assigned to the other participant. The experiment was designed and executed with the E-Prime 3 software. The stimuli consisted of a red or green square (4.5 x 4.5 cm) presented to the right or left of a fixation cross on a black background (Fig 1).

### Procedure

Participants were required to perform the joint version of the Simon task, each responding to only one stimulus, either the green or the red square, as fast and accurately as possible. For half of the sample, the red square was assigned to the left participant, the green square was assigned to the right participant, and the other half experienced the opposite stimulus-response mapping.

Each trial started with the presentation of a white fixation cross at the center of the screen. After 1 s, a colored square appeared to the left or the right of the cross until a response was emitted or for a maximum of 1 s. The intertrial interval was fixed at 1 s, during which a black screen was presented.

Participants performed 24 practice trials, which were followed by 256 experimental trials divided into two blocks of 128 trials each (64 trials for each participant). In each trial, only one of the two participants responded, hence a *go* trial for one participant was a *nogo* trial for the other. For each responding participant, in half of the trials, the stimulus appeared on the same side of the response (corresponding trials), in the other half, it appeared on the opposite side (non-corresponding trials).

After completing the task, the participants were asked if they had met their pair mate before the experiment and, if so, to specify their level of acquaintance (1 = we met once, 2 = we met few times, 3 = we met very often, 4 = we are colleagues, 5 = we are friends).

### EEG hyperscanning acquisition and data preprocessing

The neuroelectric hyperscanning recordings were performed with two synchronized 32-channel EEG acquisition systems (Brain Product GmbH, Germany), one for each participant. For each subject, data were collected through 32 active electrodes mounted on an elastic cap according to an extension of the international 10–20 system (reference on right mastoid and ground at left mastoid) and digitized to a sampling frequency of 250 Hz. Electrodes’ impedances were adjusted to values below 10 kΩ. To delete the sources of variance between the two systems, we used a calibration signal to equalize the different gains of the two amplifiers.

EEG data were processed offline using BrainVision Analyzer v.2 (Brain Product GmbH) and MATLAB v. R2020b (MathWorks). The raw EEG data were bandpass filtered in the range of 3–20 Hz to focus on the frequency content of interest for ERP analysis. Specifically, we decided to employ a high cut-off frequency higher than the one suggested in ERP studies to focus the analysis on the specific frequency content of N2 and P3 potentials (around 4 Hz), targeted in our study, thus reducing the presence of trends and drift in the EEG traces recorded [53]. Ocular correction was performed using the Gratton, Coles, and Donchin’s [54] algorithm as implemented in BrainVision Analyzer 2 using the Fpz channel as a regressor and removing it from the dataset immediately after correction. The regression method was chosen over decomposition approaches such as Independent Component Analysis for ocular artifact management, since the number of channels used in this experiment does not guarantee accurate separation of components related to ocular artifacts from those related to neuro-electrical activity. The data were then segmented into 1000 ms epochs ranging from −200–800 ms according to the time of stimulus onset. An automatic artifact rejection procedure was performed excluding all trials in which at least one channel showed an amplitude exceeding ±100 µV. As a result of this procedure, the average number of trials retained for the four conditions obtained from the combination of trial type (*go* vs. *nogo*) and correspondence (corresponding vs. non-corresponding) was as follows: *go* corresponding = 63.81; *go* non-corresponding = 63.83; *nogo* corresponding = 63.88; *nogo* non-corresponding = 63.84. A baseline correction was applied based on the 200 ms pre-stimulus. The stimulus-locked average was applied subject-wise to the different epochs separately for the four experimental conditions obtained from the combination of trial type (*go* vs. *nogo*) and correspondence (corresponding vs. non-corresponding).

At the single-brain level, the peaks of the N2 and P3 components at electrodes Fz, Cz and Pz were identified and analyzed. We limited the peak identification to these 3 electrodes since it is well-known that N2 and P3 components are not typically lateralized but, instead, have broad scalp distributions with central maxima [55]. Furthermore, previous electrophysiological investigations of the JSE focused on these three electrode sites [ 21]. To accomplish such identification, we defined two different time windows according to the stimulus onset: [200–300]ms for N2 and [300–480]ms for P3. In each of the two windows, we first identified the presence of at least one peak (in correspondence of a sign change of the first derivative of the signal) and then identified the N2 peak as the most negative peak in the time window between 200 and 300 ms, and the P3 peak as the most positive peak in the time window between 300 and 480 ms. Once the N2 and P3 peaks were identified, their main features were characterized using two simple estimators: (a) *amplitude*, defined as the absolute value of the N2/P3 voltage at its peak; and (b) *latency*, defined as the point in time at which the N2/P3 component reaches its maximum voltage before returning toward zero [55]. As for the amplitude, we adopted the peak amplitude measure, while for the latency, we used the peak latency index. We decided to use such simple estimators compared to more sophisticated ones as a balance between bias and efficiency in the ERP peak estimation process [56]. As for the N2 component, although amplitude values were expressed as absolute magnitudes to indicate the strength of the response, all peaks corresponded to negative deflections. This convention was adopted for descriptive clarity and does not affect the outcome of the statistical analyses, as all amplitudes share the same polarity.

To evaluate the temporal alignment between responding and non-responding participants, we computed the EEG-equivalent of the behavioral Joint Simon Effect (EEG-JSE) by examining the differences in the latencies between non-corresponding and corresponding trials for the N2 and P3 components, separately. More specifically, for each participant in the pair, and for the two ERP components (N2 and P3) separately, we measured the EEG-JSE by calculating the absolute difference in latencies between non-corresponding and corresponding trials. Specifically, since each trial was a *go* trial for a participant (i.e., the responding participant) and a *nogo* trial for his/her co-agent (i.e., the non-responding participant), for the responding participant, we compared the non-corresponding *go* trials with the corresponding *go* trials, while for the non-responding participant, we considered the *nogo* trials and compared the non-corresponding *nogo* trials with the corresponding *nogo* trials. By performing these calculations separately for the two participants, we were able to derive a distinct measure of the EEG-JSE for both the group of responding participants (those who were required to make a response) and the group of non-responding participants (those who did not need to respond). As shown in behavioural studies [57], the direction of the Simon effect varies across participants, and reversed effects are not uncommon. Since this variability may be present also at the ERP level, we computed the absolute difference between non-corresponding and corresponding trials. This approach prevents potential cancellation of effects when opposite signs are averaged across participants, which could otherwise obscure inter-brain alignment.

### Statistical analyses

*Behavioral data.* Six couples out of 44 reported levels of acquaintance equal or higher than 4 (see *Procedure* section). Since the results of the analyses did not change when these couples were excluded, in what follows we report the results of the analyses conducted on the whole sample.

Practice trials, errors (0.2% of the total trials), and trials that presented RTs more than 3 standard deviations below or above the participant’s overall mean (3.9% of corresponding trials and 4.1% for non-corresponding trials) were removed. The average number of retained trials was 122.94 and 122.59 for the corresponding and non-corresponding conditions, respectively.

We used R [58] and the lme4 package [59] to perform a linear mixed-effects analysis of the relationship between Correspondence and response times. The model included a fixed effect of Correspondence (corresponding vs. non-corresponding) and random intercepts for subjects nested within couples. The model specification was as follows: RT ~ Correspondence + (1|Couple/Subject). The model was fitted using maximum likelihood (ML) estimation, providing a good fit to the data (AIC = 1639.1, BIC = 1654.9, log-likelihood = –814.5). Significance of fixed effects was evaluated using a Type III ANOVA with Satterthwaite’s approximation for degrees of freedom, as implemented in the lmerTest package. To ensure interpretable main effects, sum-to-zero contrasts were applied to categorical predictors.

*Single-brain ERP analyses*. The latency and amplitude of the N2 and P3 peaks for each subject, condition, and channel were analyzed in R [58] using the lme4 [59] and lmerTest packages. Separate linear mixed-effects models were fitted for N2 amplitude, N2 latency, P3 amplitude, and P3 latency. Fixed effects included Correspondence (corresponding vs. non-corresponding), Trial Type (go vs. nogo), and Electrode (Fz, Cz, Pz), as well as all possible interactions. Random intercepts were included for subjects nested within pairs to account for repeated measures and hierarchical data structure. The model specification was as follows: dependent variable (i.e., N2 amplitude, N2 latency, P3 amplitude or P3 latency) ~ Correspondence * Trial Type * Electrode + (1 | couple/subject). Models were fitted using maximum likelihood estimation. Sum-to-zero contrasts were applied for all categorical predictors to allow for interpretable main effects. Predictors were coded using sum contrasts, with corresponding trials coded as 1 and non-corresponding trials coded as −1, *go* trials coded as 1 and *nogo* trials coded as −1. The factor Electrode (three levels) was coded using sum contrasts, with the first two levels (Electrode1/Fz and Electrode2/Cz) coded explicitly and the third level (Electrode3/Pz) coded implicitly so that the sum of coefficients across levels equals zero. Model assumptions, including normality of residuals and homoscedasticity, were inspected visually using residual plots and were deemed to be met. Significance of fixed effects was evaluated using Type III ANOVA with Satterthwaite’s approximation for degrees of freedom, yielding the reported F and p-values. Following significant main effects of Electrode and significant interactions, pairwise comparisons were corrected using the Bonferroni method. Full model estimates for each dependent variable are reported as Supporting Information (SI).

*Inter-brain ERP analyses in real versus random pairs.* To explore the extent to which the neural processing of the responding participants aligned with that of the non-responding participants, providing insight into the potential synchrony or asynchrony between the two individuals during the task, we assessed the existence of synchronization in terms of EEG-JSE between the two participants. To this end, we performed a permutation test [44,60–62], which is a statistical method that allows us to determine whether the observed effect in real data (data from real dyads recorded simultaneously during the experiment) is significantly different from what would be expected by chance (data from random dyads paired only based on the role but not simultaneously recorded). Specifically, we compared the absolute *t* value representing the temporal distance in EEG-JSEs between the two participants across *real pairs* to a distribution of values generated from 500 *random pairs* through permutation testing. These random pairs were created by randomly pairing participants who did not perform the task simultaneously, thus disrupting any temporal alignment between the individuals while maintaining the same roles of each participant (e.g., responding vs. non-responding). This procedure allowed us to evaluate how likely it was to observe the same degree of synchronization purely by chance.

The permutation procedure involved calculating the *t* values of the EEG-JSEs for each random assignment of pairs and then generating a distribution of *t* values across the 500 iterations. The real pairs’ *t* value was then compared to this distribution to assess statistical significance. If the *t* value for real pairs fell below the 5th percentile of the random pairs’ distribution (in a one-tailed test), this indicated that the EEG-JSE alignment (distance close to zero) between the two participants was significant. In other words, if the difference in N2/P3 latencies was closer to zero for the real pairs than for the random pairs, this would suggest that the real pairs exhibited a higher level of neural synchronization than would be expected by chance.

This analysis was applied separately for the N2 and P3 peak latencies, and for each electrode (Fz, Cz, and Pz) to capture the temporal dynamics of the neural responses from different regions of the scalp. By doing this, we aimed to assess whether synchronization between participants was consistent across different components of the event-related potentials (ERPs) and across different brain regions, providing a comprehensive measure of neural alignment during the task.

## Results

### Behavior

The analysis revealed a main effect of *Correspondence*, indicating a significant RT-JSE: participants responded faster in corresponding trials (M = 359 ms, SD = 53) than in non-corresponding trials (M = 369 ms, SD = 55), *F*(1, 88) = 57.90, *p* < . 0001. The model’s fixed effect estimate confirmed this difference (β = –5.04, SE = 0.66, t(88) = –7.61, *p* < .001). The random effects indicated substantial variability across participants (σ^2^ = 1357.87) and pairs (σ^2^ = 1416.40), with a residual variance of 77.29.

### Single-brain ERPs

**Fig 2:**
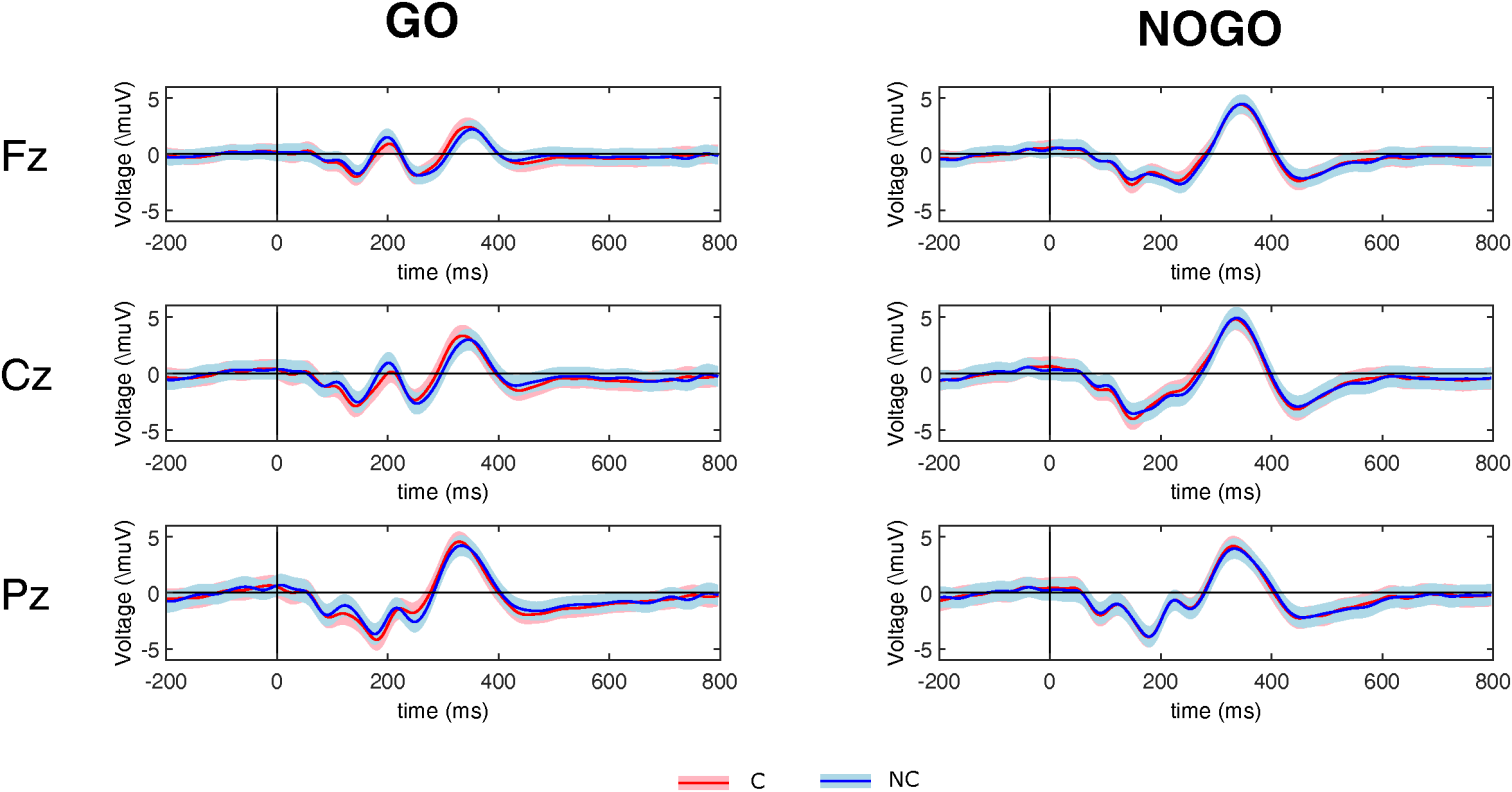
Stimulus-locked grand-average ERP waveforms. Stimulus-locked grand-average ERP waveforms at Fz, Cz, and Pz as a function of Correspondence (C vs. NC) and Trial type (go vs. nogo). C = corresponding; NC = non-corresponding. Shading represents the standard error of the mean.

**Fig 3:**
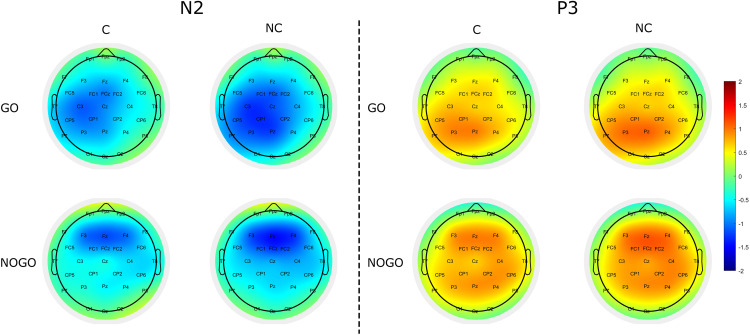
Scalp topographies. Scalp topographies of the N2 and P3 components as a function of Correspondence (C vs. NC) and Trial Type (go vs. nogo). C = corresponding; NC = non-corresponding.

Target-locked grand-average ERP waveforms at Fz, Cz, and Pz and scalp topographies of N2 and P3 as a function of Correspondence (corresponding vs. non-corresponding) and Trial type (*go* vs. *nogo*) are depicted in Figs 2 and 3, respectively. In Fig 2, we notice the presence of N2 and P3 components in both corresponding and non-corresponding trials not only in the individuals required to press the button (*go* condition) but also in the observers seated next to them (*nogo* condition). Amplitudes and latencies of the two components are comparable between the two participants in the couple.

Visual inspection of Fig 3 revealed that for both N2 and P3 components *go* trials were characterized by a centro-parietal scalp distribution, whereas *nogo* trials were mostly characterized by a frontal scalp distribution. For the N2 component, non-corresponding trials were characterized by a stronger negativity than corresponding trials in centro-parietal sites in *go* trials and in frontal sites in *nogo* trials. For the P3 component, non-corresponding trials were characterized by a stronger positivity than corresponding trials in posterior sites in *go* trials and in frontal sites in *nogo* trials.

*N2 – Latency.* Descriptive statistics for N2 latency are reported in Table 1. Type III ANOVA results are presented in Table 2. Full model estimates for each dependent variable are reported in S1 Table.

**Table 1:** Mean N2 latency (and standard deviation) in ms as a function of Electrode (Fz, Cz, Pz), Trial Type (*go* vs. *nogo*), and Correspondence (C = corresponding vs. NC = non-corresponding).

	Fz				Cz				Pz			
	Go		Nogo		Go		Nogo		Go		Nogo	
	C	NC	C	NC	C	NC	C	NC	C	NC	C	NC
**Mean**	259	263	250	251	254	257	251	248	247	250	256	256
**Sd**	19.9	19.5	23.5	21.3	18.9	19.8	26.2	23.9	18.7	19.3	21.6	23.1

**Table 2:** Type III ANOVA results (Satterthwaite) for N2 latency.

Effect	F	df_num	df_den	P
Correspondence	2.10	1	672.05	0.15
Trial Type	3.01	1	685.52	0.08
Electrode	3.59	2	674.33	0.03 *
Correspondence × Trial Type	1.15	1	677.59	0.28
Correspondence × Electrode	0.28	2	669.79	0.75
Trial Type × Electrode	18.70	2	670.15	<0.001 ***
Correspondence × Trial Type × Electrode	0.10	2	670.25	0.90

Significant codes: ‘***’ 0.001 ‘**’ 0.01 ‘*’ 0.05.

Model fit indices were as follows: AIC = 6511.1, BIC = 6580.5, and log-likelihood = –3240.6. The analysis showed a main effect of *Electrode*, *F*(2, 674.33) = 3.59, *p* = .03, and a significant *Trial type x Electrode* interaction, *F*(2, 670.15) = 18.70, *p* < .001. Even though latency was slightly longer in non-corresponding trials compared to corresponding trials, the effect of *Correspondence* did not reach significance (*p* = .09). No other main effects or interactions were significant (*p*s > .08).

**Fig 4:**
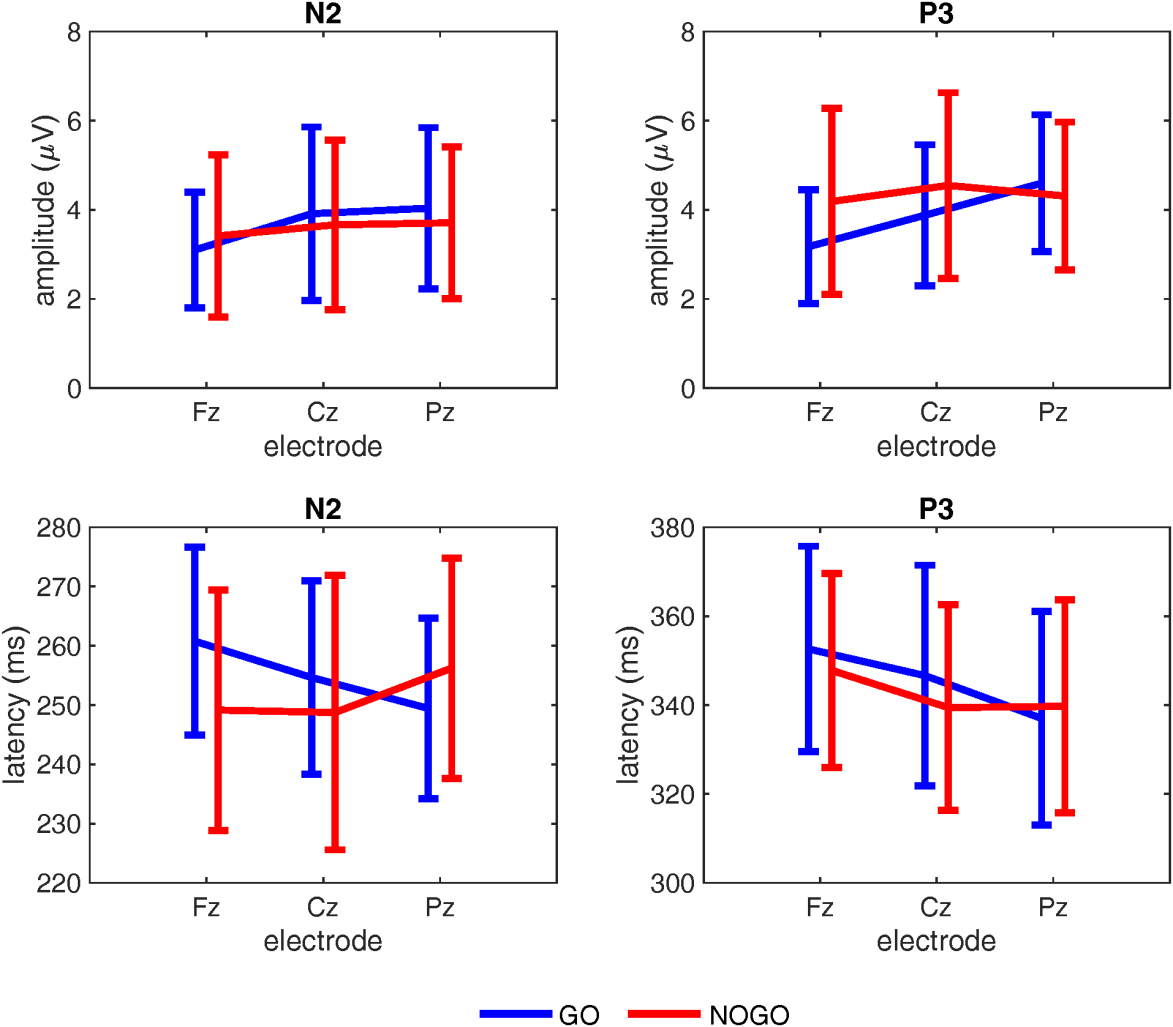
ERPs results. Mean amplitude and latency of the N2 (left panel) and P3 (right panel) components as a function of electrode site (Fz, Cz, and Pz) in *go* (blue line) and *nogo* (red line) trials. Error bars represent the standard errors of the mean.

Electrode comparisons showed only a marginal difference between Fz and Pz (estimate = 3.55, SE = 1.48, *p* = .050). Pairwise comparisons showed that in *go* trials peak latency was the longest in Fz and gradually decreased, showing significantly higher values in Fz than in Cz (estimate = 6.03, SE = 1.87, *p* = .004) and in Pz (estimate = 12.31, SE = 2.02, *p* < .001). Cz showed higher values than Pz (estimate = 6.28, SE = 2.01, *p* = .006). In *nogo* trials, peak latency was the longest in Pz, that differed from both Fz (estimate = −5.22, SE = 2.13, *p* = .044) and Cz (estimate = −5.27, SE = 2.16, *p* = .046). No difference emerged between Fz and Cz. Within-electrode comparisons revealed that Fz showed significantly higher values in *go* trials compared to *nogo* trials (estimate = 9.93, SE = 2.00, *p* < .001). The difference for Cz did not reach statistical significance (estimate = 3.95, SE = 2.04, *p* = .054). Conversely, Pz showed significantly lower values in *go* compared to *nogo* trials (estimate = −7.60, SE = 2.16, *p* < .001) (see Fig 4, left panel).

*N2 – Amplitude.* Descriptive statistics for N2 amplitude are reported in Table 3. Type III ANOVA results are presented in Table 4. Full model estimates for each dependent variable are reported in S2 Table.

**Table 3:** Mean N2 amplitude (and standard deviation) in µV as a function of Electrode (Fz, Cz, Pz), Trial Type (*go* vs. *nogo*), and Correspondence (C = corresponding vs. NC = non-corresponding). For clarity, amplitude values are reported in absolute terms to reflect the magnitude of the N2 response; however, all values refer to negative peaks. This convention does not influence the statistical results, since all amplitudes correspond to negative deflections.

	Fz				Cz				Pz			
	Go		Nogo		Go		Nogo		Go		Nogo	
	C	NC	C	NC	C	NC	C	NC	C	NC	C	NC
**Mean**	3.07	3.01	3.07	3.38	3.83	3.89	2.91	3.80	3.78	3.97	3.52	3.64
**Sd**	1.40	1.41	1.73	1.99	2.03	2.04	1.92	2.18	1.95	1.94	1.72	1.93

**Table 4:** Type III ANOVA results (Satterthwaite) for N2 amplitude.

Effect	F	df_num	df_den	P
Correspondence	6.95	1	674.71	.009 **
Trial Type	11.23	1	688.05	.001 ***
Electrode	11.55	2	677.07	<.001 ***
Correspondence × Trial Type	1.18	1	680.39	.28
Correspondence × Electrode	0.82	2	672.57	.44
Trial Type × Electrode	4.77	2	672.90	.009 **
Correspondence × Trial Type × Electrode	2.39	2	673.03	.092

Significant codes: ‘***’ 0.001 ‘**’ 0.01 ‘*’ 0.05.

Model fit indices were as follows: AIC = 2810.3, BIC = 2879.7, and log-likelihood = −1390.2. The analysis showed a main effect of *Correspondence, F*(1, 674.71)=6.95, *p = *.009, *Trial Type, F*(1, 688.05)=11.23, *p = *.001, and *Electrode, F*(2, 677.07)=11.55, *p < *.001. The *Trial Type × Electrode* interaction was significant, *F*(2, 672.90)=4.77, *p* = .009, while other interactions, including the three-way interaction, were not significant (*p*s > .09). Corresponding trials elicited smaller amplitudes than non-corresponding trials (estimate = −0.27, SE = 0.10, *p* = .009), and *go* trials produced larger amplitudes than *nogo* trials (estimate = 0.35, SE = 0.11, *p* = .001). Electrode comparisons indicated that Fz had lower amplitudes than Cz (estimate = −0.48, SE = 0.12, *p* < .001) and Pz (estimate = −0.54, SE = 0.13, *p* < .001). No difference emerged between Cz and Pz. The *Trial Type × Electrode* interaction showed that during *go* trials, amplitudes at Fz were significantly lower than at Cz (estimate = −0.86, SE = 0.16, *p* < .0001) and Pz (estimate = −0.74, SE = 0.17, *p* < .001), while no difference emerged between Cz and Pz. No significant differences were observed across electrodes in *nogo* trials. At the within-electrode level, the difference in peak amplitude between *go* and *nogo* trials was significant only at the Cz (estimate = 0.71. SE = 0.18, *p* < .001) and Pz (estimate = 0.37, SE = 0.19, *p* = .05) (see Fig 4, left panel).

*P3 - Latency*. Descriptive statistics for P3 latency are reported in Table 5. Type III ANOVA results are presented in Table 6. Full model estimates for each dependent variable are reported in S3 Table.

**Table 5:** Mean P3 latency (and standard deviation) in ms as a function of Electrode (Fz, Cz, Pz), Trial Type (*go* vs. *nogo*), and Correspondence (C = corresponding vs. NC = non-corresponding).

	Fz				Cz				Pz			
	Go		Nogo		Go		Nogo		Go		Nogo	
	C	NC	C	NC	C	NC	C	NC	C	NC	C	NC
**Mean**	355	355	348	350	346	348	339	341	334	340	337	342
**Sd**	27.7	23.6	23.1	25.1	28.8	26.8	24.6	27.9	24.8	28.3	27.4	27.8

**Table 6:** Type III ANOVA results (Satterthwaite) for P3 latency.

Effect	F	df_num	df_den	P
Correspondence	9.09	1	888.65	.003 **
Trial Type	9.46	1	889.43	.002**
Electrode	60.21	2	888.44	<.001 ***
Correspondence × Trial Type	0.41	1	888.91	.52
Correspondence × Electrode	1.17	2	888.23	.31
Trial Type × Electrode	7.45	2	888.39	<.001 ***
Correspondence × Trial Type × Electrode	0.13	2	888.32	.88

Significant codes: ‘***’ 0.001 ‘**’ 0.01 ‘*’ 0.05.

Model fit indices were as follows: AIC = 8559.9, BIC = 8633.1, and log-likelihood = −4264.9. The analysis revealed significant main effects of *Correspondence*, *F*(1, 888.65) = 9.09, *p* = .003, *Trial type*, *F*(1, 889.43) = 9.46, *p* = .002, and *Electrode*, *F*(2, 888.44) = 60.21, *p* < .001, and a significant *Trial type* x *Electrode* interaction, *F*(2, 888.39) =7.45, *p* < .001. Peak latency was shorter in corresponding trials compared to non-corresponding trials (estimate = −3.26, SE = 1.09, *p* = .003) and longer in *go* than in *nogo* trials (estimate = 3.34, SE = 1.09, *p* = .002). Peak latency was significantly longer at Fz compared to Cz (estimate = 9.11, SE = 1.33, *p* < .0001) and Pz (estimate = 14.45, SE = 1.34, *p* < .0001), and longer at Cz than Pz (estimate = 5.34, SE = 1.32, *p* = .0002). Pairwise comparisons revealed significant differences across all electrodes, with peak latency gradually decreasing from Fz, where it was the highest, to Pz, where it was the lowest in *go* trials (all *ps < *.001). In *nogo* trials, latency was significantly higher in Fz compared to Cz (estimate = 9.58, SE = 1.88, *p < *.0001) and Pz (estimate = 10.29, SE = 1.90, *p < *.0001), whereas no significant difference was found between Cz and Pz. Within electrodes, the difference between *go* and *nogo* trials was significant only for Fz (estimate = 5.80, SE = 1.91, *p = *.003), and Cz (estimate = 6.74, SE = 1.87, *p < *.001).

*P3 – Amplitude.* Descriptive statistics for P3 amplitude are reported in Table 7. Type III ANOVA results are presented in Table 8. Full model estimates for each dependent variable are reported in S4 Table.

**Table 7:** Mean P3 amplitude (and standard deviation) in µV as a function of Electrode (Fz, Cz, Pz), Trial Type (*go* vs. *nogo*), and Correspondence (C = corresponding vs. NC = non-corresponding).

	Fz				Cz				Pz			
	Go		Nogo		Go		Nogo		Go		Nogo	
	C	NC	C	NC	C	NC	C	NC	C	NC	C	NC
**Mean**	3.15	2.99	3.99	4.11	3.80	3.84	4.24	4.67	4.52	4.62	4.20	4.19
**Sd**	1.38	1.39	2.07	2.29	1.70	1.70	2.09	2.25	1.61	1.71	1.77	1.70

**Table 8:** Type III ANOVA results (Satterthwaite) for P3 amplitude.

Effect	F	df_num	df_den	P
Correspondence	1.32	1	899.11	.25
Trial Type	27.97	1	890.06	<.001 ***
Electrode	42.49	2	888.86	<.001 ***
Correspondence × Trial Type	1.19	1	889.46	.27
Correspondence × Electrode	0.73	2	888.60	.48
Trial Type × Electrode	31.61	2	888.82	<.001***
Correspondence × Trial Type × Electrode	0.84	2	888.71	.43

Significant codes: ‘***’ 0.001 ‘**’ 0.01 ‘*’ 0.05.

The full model produced a singular fit, indicating that one or more variance components were estimated as zero and could not be reliably identified given the available data. To ensure stable and interpretable variance estimates, we simplified the random effects structure to include only random intercepts for subjects. Model fit indices were as follows: AIC = 3449.1, BIC = 3517.4, and log-likelihood = −1710.5. The analysis showed significant main effects of *Trial type*, *F*(1, 890.06) = 27.97, *p* < .001, and *Electrode, F*(2, 888.86) = 42.49, *p* < .001, and a significant interaction between *Trial type* and *Electrode*, *F*(2, 888.82) = 31.61, *p* < .001. Amplitude was smaller in *go* than in *nogo* trials (estimate = −0.43, SE = 0.08, *p < .0001),* and in Fz than in Cz (estimate = −0.64, SE = 0.09, *p* < .001) and Pz (estimate = −0.87, SE = 0.10, *p* < .001). No significant difference emerged between Cz and Pz (estimate = −0.23, SE = 0.10*, p* = .06). In *go* trials, all electrodes differed between each other (*p*s < .0001). In *nogo* trials, Fz differed from Cz (estimate = −0.45, SE = 0.14, *p* = .004), while no difference emerged between Fz and Pz and between Cz and Pz. Within-electrode comparisons showed that the difference between *go* and *nogo* trials was significant at all three electrodes (*p*s <=.001), but while in both Fz and Cz electrodes amplitude was lower in *go* than in *nogo* trials (Fz: estimate = −1.05, SE = 0.14, Cz: estimate = −0.67, SE = 0.14), in Pz it was higher in *go* than in *nogo* trials (estimate = 0.45, SE = 0.14) (see Fig 4, right panel).

#### Inter-brain ERPs in real versus random pairs.

**Fig 5:**
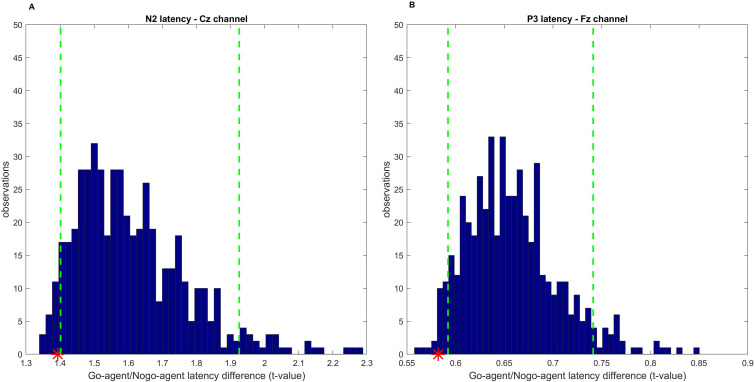
Permutation test results. Distribution of *t* values representing the absolute temporal distance between EEG-JSEs for two paired participants, created under the null hypothesis (500 random pairs created via permutation testing, while preserving each participant’s role). Red asterisks indicate the absolute *t* values obtained for the real pairs. Green dotted lines represent the 5^th^ percentile thresholds. Results are shown for the N2 component at electrode Cz (panel A) and the P3 component at electrode Fz (panel B).

The results of the permutation test applied to the EEG-JSEs shown by each pair of participants (real pairs) revealed a synchronization between the responding and non-responding participants, specifically for the N2 component at the Cz electrode and for the P3 component at the Fz electrode, as shown in Fig 5. Notably, for these electrodes only, the temporal discrepancy in EEG-JSEs between the responding and non-responding participants of real pairs was below the 5^th^ percentile threshold of the random distribution (indicating a minimal difference, close to zero). The permutation test did not provide any significant result for the other electrode positions. This result demonstrated a significant temporal alignment in the brain responses, as recorded from the Cz (for the N2 component) and Fz (for the P3 component) electrode sides, of real pairs compared to random pairs, supporting the hypothesis of interlinked cognitive processing during task execution.

## General discussion

In the present study, we aimed to assess whether during performance of the Joint Simon task participants in a dyad showed synchronized brain activity, considering this synchronization as an index of self-other integration. Specifically, we assessed whether the EEG-JSE, that is the neural counterpart of the behavioural JSE, computed for the two interacting participants were synchronized. Overall, we found a significant JSE at the behavioral level. As regards the neural signatures, single-brain ERP analyses indicated that both the N2 and P3 components were modulated by response conflict, with an effect of correspondence emerging for N2 amplitude and P3 latency. Crucially, ERP data from real pairs showed inter-brain synchronization: both the N2-JSE and the P3-JSE were aligned across pairs at the Cz (N2) and Fz (P3) electrodes. In the sections that follow, we elaborate on these results.

### Behavior

The analysis of the behavioural data revealed a significant 10-ms JSE, with faster RTs in corresponding trials than in non-corresponding trials. The magnitude of the effect is consistent with that found in previous studies using a similar paradigm [51,63].

### Single-brain ERPs

Consistent with prior research, we focused our single-brain analyses on the N2 and P3 ERP components, as these have been shown to be modulated by the requirement to perform the Simon task jointly [20,21]. However, in contrast to previous studies, our focus was on how neural responses—specifically peak latency and amplitude—are modulated by trial type and correspondence within a social context.

#### N2 latency and amplitude.

Our analyses revealed differences between trial types in both peak latency and amplitude, with distinct distributions across the scalp and the three electrodes. The finding of higher N2 amplitude and longer latency in *go* trials compared to *nogo* trials is consistent with the results of previous studies [64]. Since this component is thought to reflect conflict monitoring, detection, and inhibition in general and inhibition of response preparation in specific [20,22], the difference between *go* and *nogo* trials may indicate that conflict, affecting both processing speed and required resources, is greater when a response is executed [64].

Correspondence affected N2 amplitude, with larger amplitude in non-corresponding trials than in corresponding trials. The effect of correspondence was largely consistent across trial types, with no strong evidence of moderation by trial type. It is important to note that the finding of an effect of correspondence also on *nogo* N2 amplitude may indicate that, in the non-responding participant, non-corresponding trials were characterized by higher conflict compared to corresponding trials. Crucially, since correspondence is defined for the responding agent (*go* trials), in a *nogo* non-corresponding trial the stimulus is presented on the side of the non-responding agent. This stimulus may hence induce in this agent a stronger propensity to respond and thus a higher conflict as compared to a stimulus that is presented on the contralateral side (i.e., the responding participant’s side) [65]. Alternatively, this higher conflict may be due to a vicarious experience of a conflict, that is a conflict induced by observing the conflict experienced by the co-agent [66]. While our data alone do not allow us to disentangle among these two alternative explanations, they seem to suggest that the behavioral JSE, which reflects a difference in cognitive processing between corresponding and non-corresponding stimuli, may emerge at the electrophysiological level also for the non-responding participant. It should be noted that Tsai et al. [21] found an effect of correspondence on N2 amplitude in *nogo* trials. However, this effect did not differ between the joint and the individual *go*-*nogo* versions of the Simon task, hence suggesting that conflict monitoring, detection and inhibition in *nogo* trials may occur irrespective of whether the task is performed individually or jointly. Since we did not assess differences between social and non-social contexts, our results should hence be taken with caution.

#### P3 latency and amplitude.

As regards the P3 component, our analyses revealed a sensitivity of P3 latency to trial type, with higher latency in *go* trials as compared to *nogo* trials, in line with what was found for the N2 latency. Furthermore, an effect of correspondence was evident, with a slower P3 in non-corresponding than in corresponding trials.

The amplitude of the P3 component was significantly larger in *nogo* trials than in *go* trials. Such a result is in line with the finding of a larger *nogo*-P3 during the JST compared to the individual *go*-*nogo* task [18–21] and may reflect the need to inhibit the tendency to respond when it is their co-actor’s turn [20,21]. Crucially, modulations of the *nogo*-P3 by the social context was not evident in patients with schizophrenia performing a JST [18]. According to the authors, these patients, which are known for having severe deficits in a wide variety of socio-cognitive processes [67], do not co-represent the action of their co-actor and therefore, in *nogo* trials, they do not need to exert more control.

Even though the results of the present study seem to suggest that self-other integration processes at the basis of the JSE may reflect on the modulations of the N2 and P3 components generated during joint action in both the responding and non-responding participants, they are not conclusive and are open to different interpretations. Stronger support to the idea that joint action entails self-other integration comes from the inter-brain ERP analyses presented in the following section.

### Inter-brain ERPs of real vs. random pairs

In this study, we used an EEG hyperscanning approach to investigate the eventual synchronized brain responses between responding and non-responding participants, which we considered as an indication of self-other integration during the performance of the JST. As predicted, we found a significant synchronization between the EEG-JSE of real pairs: the N2-JSE and the P3-JSE were aligned at the Cz and Fz electrodes, respectively. This alignment is in line with the idea that joint action entails self-other integration processes [3,68,69]. Most importantly, our results complement those obtained in previous fNIRS-based hyperscanning studies [48] by showing that self-other integration processes, emerging during joint performance, may reflect not only in the interpersonal neural synchronization evident in frontal regions but also in a synchronization between the EEG-JSE.

We suggest that the evidence of an alignment between the EEG-JSEs of the two participants found in the present study may indicate that the social setting affects action representations. That is, although it seems established that the JSE may emerge in non-social settings because of a non-socially induced spatial coding processing [70], our results are coherent with those of the studies that investigated the fractal structure and temporal correlation of RTs of individuals performing the JST and demonstrated that sharing a task with a co-agent entails inter-individual dynamics that are not present when acting in isolation [71,72]. Importantly, the results of the present study show that these joint dynamics can be measured not only at the behavioral level, as reflected by the coupling of co-agents’ behavior [73], but also at the neural level, as shown by the coupling of co-agents EEG-JSEs. In our analysis, however, we evaluated the EEG-JSE as the absolute difference between the latency of corresponding and non-corresponding trials for both the N2 and P3 components, as this approach allowed us to capture the overall magnitude of the effect, regardless of whether the latencies were faster in corresponding or non-corresponding trials. However, we fully acknowledge that the direction of these latency differences—whether the latencies are faster for corresponding or non-corresponding trials—could offer additional insights into the underlying neural processes. The specific pattern of latency differences may reflect different cognitive strategies or interactions between the participants and exploring this could lead to a more nuanced understanding of the JSE at the neural level. As such, we agree that future work should address this limitation by explicitly investigating the directionality of latency differences and its potential implications for the interpretation of inter-brain synchrony during joint tasks.

We are aware that the social vs. nonsocial nature of the JSE is still debated. Previous investigations of the JSE were non-conclusive regarding the social or non-social nature of the effect, with some studies stressing the modulation of the effect by social factors [73] and others demonstrating that the JSE can be elicited also in the absence of a human co-agent [74,75]. However, the exploration of inter-brain neural dynamics (as allowed by a hyperscanning setup) brings a new perspective on joint action that can potentially account for the complexity of social interactions which is not entirely accessible through the investigation of isolated minds [36,76]. It is important to underline that, given the alternation of responses required by the JST, individuals would perform better by ignoring each other. Nonetheless, the human brain seems to automatically adjust to the presence of another individual performing half of the task [14,15]. The present investigation suggests that the tendency to implicitly coordinate with others, even when not explicitly requested, is accompanied by complex inter-brain dynamics which take place at the cost of a decline in individual performance.

## Conclusion

Single-brain data allowed us to describe the modulation of the N2 and P3 components by trial type and correspondence. However, these results do not allow to unveil the specific and complex dynamics supporting joint action. Our novel EEG hyperscanning approach to ERPs tried to overstep these limitations by allowing for the assessment of the interaction between the brains of the dyads jointly performing a task. Even if a causal interpretation of ERP synchronization cannot be assumed [44], our findings might represent the tip of the iceberg of the complex inter-brain dynamics uniquely associated with the self-other integration process emerging during joint action. Future investigations might benefit from this approach by addressing the low-level mechanisms supporting self-other integration during joint action.

One of the key contributions of this study is the introduction of the EEG-JSE as a novel metric for measuring inter-brain synchronization during joint action, which may allow us to capture the neural alignment between both participants. The results of our permutation test, showing that the EEG-JSE alignment was significantly stronger in real pairs compared to random pairs, further validate this measure as an index of shared neural processing.

Our analyses were not free of limitations. First, we focused on the Fz, Cz, and Pz electrodes where the N2 and P3 components are more evident, thus not exploring other areas. Second, our sample was mostly composed of female participants and same-gender pairs. Future studies should include in the analyses larger portions of the brain and with "test more gender-balanced samples.

To conclude, the results of the present study suggest that the need to perform a task jointly may lead to unique inter-brain interaction dynamics. We believe that the methodology applied in this study to investigate the JSE offers a promising approach that can help us identify the brain processing underlying social interactions and joint action.

## Supporting information

S1 Table Linear mixed-effects model estimates for N2 latency with Correspondence (corresponding vs. non-corresponding), Trial Type (go vs. nogo), and Electrode (Fz, Cz, and Pz) as fixed effects. Estimates represent deviations from the grand mean due to sum-to-zero contrasts. The table shows fixed-effect estimates, 95% confidence intervals (CI), and p-values. Significant effects are highlighted in bold (*p* < .05). Random effects include intercepts for couples and subjects nested within couples. Variance components (σ^2^, τ₀₀), intra-class correlation (ICC), sample size, and marginal/conditional R^2^ are also reported.
(DOCX)

S2 Table Linear mixed-effects model estimates for N2 amplitude with Correspondence (corresponding vs. non-corresponding), Trial Type (go vs. nogo), and Electrode (Fz, Cz, and Pz) as fixed effects. Estimates represent deviations from the grand mean due to sum-to-zero contrasts. The table shows fixed-effect estimates, 95% confidence intervals (CI), and p-values. Significant effects are highlighted in bold (*p* < .05). Random effects include intercepts for couples and subjects nested within couples. Variance components (σ^2^, τ₀₀), intra-class correlation (ICC), sample size, and marginal/conditional R^2^ are also reported.
(DOCX)

S3 Table Linear mixed-effects model estimates for P3 latency with Correspondence (corresponding vs. non-corresponding), Trial Type (go vs. nogo), and Electrode (Fz, Cz, and Pz) as fixed effects. Estimates represent deviations from the grand mean due to sum-to-zero contrasts. The table shows fixed-effect estimates, 95% confidence intervals (CI), and p-values. Significant effects are highlighted in bold (*p* < .05). Random effects include intercepts for couples and subjects nested within couples. Variance components (σ^2^, τ₀₀), intra-class correlation (ICC), sample size, and marginal/conditional R^2^ are also reported.
(DOCX)

S4 Table Linear mixed-effects model estimates for P3 amplitude with Correspondence (corresponding vs. non-corresponding), Trial Type (go vs. nogo), and Electrode (Fz, Cz, and Pz) as fixed effects. Estimates represent deviations from the grand mean due to sum-to-zero contrasts. The table shows fixed-effect estimates, 95% confidence intervals (CI), and p-values. Significant effects are highlighted in bold (*p* < .05). Random effects include intercepts for subjects. Variance components (σ^2^, τ₀₀), intra-class correlation (ICC), sample size, and marginal/conditional R^2^ are also reported.
(DOCX)
