# Supplementary material for: Inter-brain ERPs alignment during a joint Simon task: An EEG hyperscanning study
Source: PLoS One. 2026 Jan 8;21(1):e0338934. doi: 10.1371/journal.pone.0338934 (PMC12782412; doi:10.1371/journal.pone.0338934)
Supplement: S1 Table — Estimates represent deviations from the grand mean due to sum-to-zero contrasts. The table shows fixed-effect estimates, 95% confidence intervals (CI), and p-values. Significant effects are highlighted in bold (p < .05). Random effects include intercepts for couples and subjects nested within couples. Variance components (σ2, τ₀₀), intra-class correlation (ICC), sample size, and marginal/conditional R2 are also reported. (DOCX) [file pone.0338934.s001.docx]

**S1 Table.**

|  | **Latency_N2** | | |
| --- | --- | --- | --- |
| *Predictors* | *Estimates* | *CI* | *P* |
| (Intercept) | 254.47 | 250.86 – 258.09 | **<0.001** |
| Correspondence1 | -0.85 | -2.01 – 0.30 | 0.148 |
| Trial Type1 | 1.05 | -0.14 – 2.23 | 0.083 |
| Electrode [1] | 2.20 | 0.58 – 3.81 | **0.008** |
| Electrode [2] | -0.85 | -2.46 – 0.77 | 0.306 |
| Correspondence1 × Trial Type1 | -0.63 | -1.80 – 0.53 | 0.285 |
| Correspondence1 × Electrode [1] | -0.49 | -2.09 – 1.11 | 0.548 |
| Correspondence1 × Electrode [2] | 0.56 | -1.05 – 2.17 | 0.495 |
| Trial Type1 × Electrode [1] | 3.92 | 2.32 – 5.52 | **<0.001** |
| Trial Type1 × Electrode [2] | 0.93 | -0.69 – 2.54 | 0.260 |
| (Correspondence1 × Trial Type1) × Electrode [1] | 0.04 | -1.56 – 1.64 | 0.964 |
| (Correspondence1 × Trial Type1) × Electrode [2] | -0.35 | -1.96 – 1.27 | 0.674 |
| **Random Effects** | | | |
| σ^2^ | 250.35 | | |
| τ_00_ _subject:couple_ | 158.06 | | |
| τ_00_ _couple_ | 51.79 | | |
| ICC | 0.46 | | |
| N _subject_ | 87 | | |
| N _couple_ | 44 | | |
| Observations | 754 | | |
| Marginal R^2^ / Conditional R^2^ | 0.039 / 0.477 | | |
