# Supplementary material for: Inter-brain ERPs alignment during a joint Simon task: An EEG hyperscanning study
Source: PLoS One. 2026 Jan 8;21(1):e0338934. doi: 10.1371/journal.pone.0338934 (PMC12782412; doi:10.1371/journal.pone.0338934)
Supplement: S2 Table — Estimates represent deviations from the grand mean due to sum-to-zero contrasts. The table shows fixed-effect estimates, 95% confidence intervals (CI), and p-values. Significant effects are highlighted in bold (p < .05). Random effects include intercepts for couples and subjects nested within couples. Variance components (σ2, τ₀₀), intra-class correlation (ICC), sample size, and marginal/conditional R2 are also reported. (DOCX) [file pone.0338934.s002.docx]

**S2 Table.**

|  | **Amplitude_N2** | | |
| --- | --- | --- | --- |
| *Predictors* | *Estimates* | *CI* | *p* |
| (Intercept) | 3.40 | 3.08 – 3.72 | **<0.001** |
| Correspondence1 | -0.13 | -0.23 – -0.03 | **0.009** |
| Trial Type1 | 0.17 | 0.07 – 0.28 | **0.001** |
| Electrode [1] | -0.34 | -0.48 – -0.20 | **<0.001** |
| Electrode [2] | 0.14 | 0.00 – 0.28 | **0.043** |
| Correspondence1 × Trial Type1 | 0.06 | -0.04 – 0.16 | 0.279 |
| Correspondence1 × Electrode [1] | 0.07 | -0.07 – 0.21 | 0.318 |
| Correspondence1 × Electrode [2] | -0.08 | -0.22 – 0.06 | 0.242 |
| Trial Type1 × Electrode [1] | -0.19 | -0.33 – -0.05 | **0.007** |
| Trial Type1 × Electrode [2] | 0.18 | 0.04 – 0.32 | **0.011** |
| (Correspondence1 × Trial Type1) × Electrode [1] | -0.00 | -0.14 – 0.13 | 0.950 |
| (Correspondence1 × Trial Type1) × Electrode [2] | 0.14 | 0.00 – 0.28 | **0.050** |
| **Random Effects** | | | |
| σ^2^ | 1.85 | | |
| τ_00_ _subject:couple_ | 1.05 | | |
| τ_00_ _couple_ | 0.50 | | |
| ICC | 0.46 | | |
| N _subject_ | 87 | | |
| N _couple_ | 44 | | |
| Observations | 754 | | |
| Marginal R^2^ / Conditional R^2^ | 0.043 / 0.480 | | |
