# Supplementary material for: Inter-brain ERPs alignment during a joint Simon task: An EEG hyperscanning study
Source: PLoS One. 2026 Jan 8;21(1):e0338934. doi: 10.1371/journal.pone.0338934 (PMC12782412; doi:10.1371/journal.pone.0338934)
Supplement: S3 Table — Estimates represent deviations from the grand mean due to sum-to-zero contrasts. The table shows fixed-effect estimates, 95% confidence intervals (CI), and p-values. Significant effects are highlighted in bold (p < .05). Random effects include intercepts for couples and subjects nested within couples. Variance components (σ2, τ₀₀), intra-class correlation (ICC), sample size, and marginal/conditional R2 are also reported. (DOCX) [file pone.0338934.s003.docx]

**S3 Table**.

|  | **Latency_P3** | | |
| --- | --- | --- | --- |
| *Predictors* | *Estimates* | *CI* | *p* |
| (Intercept) | 344.94 | 340.29 – 349.59 | **<0.001** |
| Correspondence1 | -1.63 | -2.69 – -0.57 | **0.003** |
| Trial Type1 | 1.67 | 0.60 – 2.73 | **0.002** |
| Electrode [1] | 7.85 | 6.34 – 9.36 | **<0.001** |
| Electrode [2] | -1.26 | -2.75 – 0.23 | 0.098 |
| Correspondence1 × Trial Type1 | -0.35 | -1.41 – 0.72 | 0.521 |
| Correspondence1 × Electrode [1] | 0.87 | -0.64 – 2.38 | 0.260 |
| Correspondence1 × Electrode [2] | 0.25 | -1.24 – 1.74 | 0.742 |
| Trial Type1 × Electrode [1] | 1.23 | -0.28 – 2.74 | 0.110 |
| Trial Type1 × Electrode [2] | 1.70 | 0.21 – 3.19 | **0.026** |
| (Correspondence1 × Trial Type1) × Electrode [1] | 0.39 | -1.12 – 1.90 | 0.612 |
| (Correspondence1 × Trial Type1) × Electrode [2] | -0.15 | -1.65 – 1.34 | 0.839 |
| **Random Effects** | | | |
| σ^2^ | 282.97 | | |
| τ_00_ _subject:couple_ | 370.85 | | |
| τ_00_ _couple_ | 48.19 | | |
| ICC | 0.60 | | |
| N _subject_ | 88 | | |
| N _couple_ | 44 | | |
| Observations | 976 | | |
| Marginal R^2^ / Conditional R^2^ | 0.061 / 0.622 | | |
