# Supplementary material for: Inter-brain ERPs alignment during a joint Simon task: An EEG hyperscanning study
Source: PLoS One. 2026 Jan 8;21(1):e0338934. doi: 10.1371/journal.pone.0338934 (PMC12782412; doi:10.1371/journal.pone.0338934)
Supplement: S4 Table — Estimates represent deviations from the grand mean due to sum-to-zero contrasts. The table shows fixed-effect estimates, 95% confidence intervals (CI), and p-values. Significant effects are highlighted in bold (p < .05). Random effects include intercepts for subjects. Variance components (σ2, τ₀₀), intra-class correlation (ICC), sample size, and marginal/conditional R2 are also reported. (DOCX) [file pone.0338934.s004.docx]

**S4 Table.**

|  | **Amplitude_P3** | | |
| --- | --- | --- | --- |
| *Predictors* | *Estimates* | *CI* | *P* |
| (Intercept) | 3.95 | 3.66 – 4.24 | **<0.001** |
| Correspondence1 | -0.05 | -0.12 – 0.03 | 0.250 |
| Trial Type1 | -0.21 | -0.29 – -0.13 | **<0.001** |
| Elecrode [1] | -0.51 | -0.62 – -0.39 | **<0.001** |
| Electrode [2] | 0.14 | 0.03 – 0.25 | **0.014** |
| Correspondence1 × Trial Type1 | 0.04 | -0.03 – 0.12 | 0.275 |
| Correspondence1 × Electrode [1] | 0.05 | -0.06 – 0.17 | 0.332 |
| Correspondence1 × Electrode [2] | -0.06 | -0.17 – 0.05 | 0.269 |
| Trial Type1 × Electrode [1] | -0.32 | -0.43 – -0.20 | **<0.001** |
| Trial Type1 × Electrode [2] | -0.12 | -0.23 – -0.01 | **0.035** |
| (Correspondence1 × Trial Type1) × Electrode [1] | 0.03 | -0.08 – 0.14 | 0.567 |
| (Correspondence1 × Trial Type1) × Electrode [2] | 0.04 | -0.07 – 0.15 | 0.472 |
| **Random Effects** | | | |
| σ^2^ | 1.54 | | |
| τ_00_ _subject_ | 1.80 | | |
| ICC | 0.54 | | |
| N _subject_ | 88 | | |
| Observations | 976 | | |
| Marginal R^2^ / Conditional R^2^ | 0.079 / 0.576 | | |
